# Dehydroabietic Acid Is a Novel Survivin Inhibitor for Gastric Cancer

**DOI:** 10.3390/plants10061047

**Published:** 2021-05-22

**Authors:** Won-Jin Kim, Woong Kim, Jang-Mi Bae, Jungsoo Gim, Seok-Jun Kim

**Affiliations:** 1 Department of Integrative Biological Sciences, Chosun University, Gwangju 61452, Korea; wjsh003@naver.com (W.-J.K.); roses987@hanmail.net (J.-M.B.); jgim@chosun.ac.kr (J.G.); 2 BK21 FOUR Educational Research Group for Age-Associated Disorder Control Technology, Chosun University, Gwangju 61452, Korea; gadak2@naver.com; 3 GARD Cohort Research Center, Department of Biomedical Science, Chosun University, Gwangju 61452, Korea

**Keywords:** dehydroabietic acid, gastric cancer, survivin

## Abstract

Gastric cancer is a malignant tumor with a high incidence and mortality rate worldwide. Nevertheless, anticancer drugs that can be used for gastric cancer treatment are limited. Therefore, it is important to develop targeted anticancer drugs for the treatment of gastric cancer. Dehydroabietic acid (DAA) is a diterpene found in tree pine. Previous studies have demonstrated that DAA inhibits gastric cancer cell proliferation by inducing apoptosis. However, we did not know how DAA inhibits the proliferation of gastric cancer cells through apoptosis. In this study, we attempted to identify the genes that induce cell cycle arrest and cell death, as well as those which are altered by DAA treatment. DAA-regulated genes were screened using RNA-Seq and differentially expressed genes (DEGs) analysis in AGS cells. RNA-Seq analysis revealed that the expression of survivin, an apoptosis inhibitor, was significantly reduced by DAA treatment. We also confirmed that DAA decreased survivin expression by RT-PCR and Western blotting analysis. In addition, the ability of DAA to inhibit survivin was compared to that of YM-155, a known survivin inhibitor. DAA was found to have a stronger inhibitory effect in comparison with YM-155. DAA also caused an increase in cleaved caspase-3, an apoptosis-activating protein. In conclusion, DAA is a potential anticancer agent for gastric cancer that inhibits survivin expression.

## 1. Introduction

Cancer is a disease wherein cells continuously divide due to an abnormal cell cycle. Among of various forms of cancer, gastric cancer was reported to have 1 million new cases and approximately 769,000 deaths in 2020 [1,2]. Commonly used cancer therapies include surgery, chemotherapy, and radiation therapy. Among these, cytotoxic anticancer drugs are mainly used for treating advanced gastric cancer [3,4]. However, since cytotoxic anticancer drugs can damage normal tissues, it is important to target only the genes of cancer cells [5].

Diterpene, a chain derivative consisting of 20 carbon atoms among terpenes, is the main component of plant essential oils [6]. They are known to have antimicrobial and anti-inflammatory effects [7]. Dehydroabietic acid (DAA) is a diterpene. In our previous study, DAA-induced cell cycle arrest and apoptosis induction in gastric cancer were demonstrated [8]. In this study, we tried to determine the genes regulated by DAA that inhibit the proliferation of gastric cancer cells.

Types of cell death include programmed cell death, autophagic cell death, and necrosis. Necrotic cell death is caused by external factors, such as infection or toxins. Cell death caused by these factors induces an inflammatory response [9,10]. In the tumor microenvironment, tumor growth is sometimes promoted by such inflammation. As a result, there is an increase in cancer cell proliferation, metastasis, and invasion [11]. Apoptosis is a process of programmed cell death. Compared to necrosis, apoptosis does not cause damage in vivo [12,13]. Therefore, induction of apoptosis can be applied to cancer therapy.

Survivin is a member of the inhibitor of apoptosis (IAP) family. In addition, survivin is encoded by *BIRC5*. Survivin negatively regulates programmed cell death or apoptosis by inhibiting caspase activation [14,15]. Inhibition of caspase activity helps cancer cells survive by interfering with apoptotic signals [16]. Moreover, the expression level of survivin correlates with poor clinical outcomes and more aggressive disease [17].

Survivin is a well-known therapeutic target for cancer. Despite being discovered 20 years ago, survivin remains a major target in cancer research [18]. Therefore, in this study, we tried to find out the genes that regulate the proliferation and apoptosis of gastric cancer by DAA treatment. We identified the DAA target gene, which was determined in a previous study. RNA-Seq was performed to identify the gene that inhibits the proliferation of gastric cancer cells. In addition, IAP family member survivin was discovered through DEG analysis, and it was suggested that DAA is a specific inhibitor of survivin.

## 2. Results

### 2.1. DAA Significantly Reduced the Growth Rate of Gastric Cancer Cells

**Figure 1:**
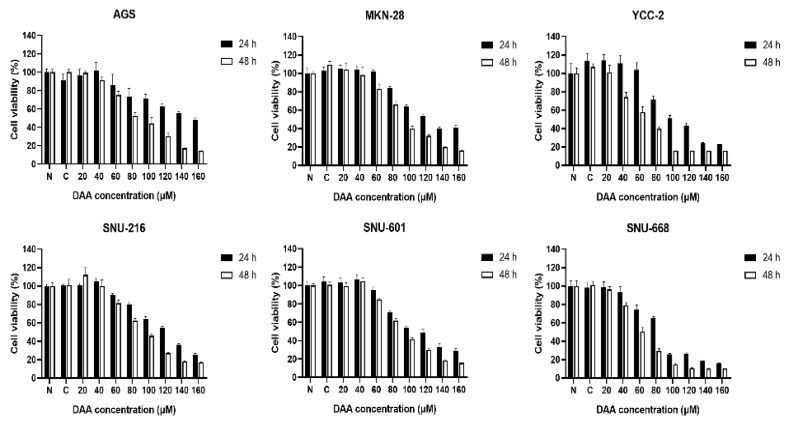
DAA inhibited the cell growth in gastric cancer cells. WST-8 assays were performed to detect the cell viability by DAA treatment for 24 and 48 h in six gastric cancer cell lines (AGS, MKN-28, YCC-2, SNU-216, SNU-601, and SNU-668). Data are presented as mean ± SEM (*n* = 5). The statistical analysis was carried out by two-way ANOVA, *p* < 0.0001.

We performed a cell proliferation assay to determine whether DAA inhibited the proliferation of gastric cancer cells. Gastric cancer cell lines (AGS, MKN-28, YCC-2, SNU-216, SNU-601, and SNU-668) were treated with different concentrations of DAA. Cell viability was determined after 24 and 48 h of incubation. A dose-dependent decrease was observed in all gastric cancer cell lines (Figure 1). Nevertheless, it showed a higher growth inhibition rate at 48 h than at 24 h after treatment with DAA. These data indicate that DAA inhibited the viability of gastric cancer cells in a time- and dose-dependent manner.

### 2.2. DAA Treatment in AGS Cells Modulated Gene Expression Related to Cell Proliferation

**Figure 2:**
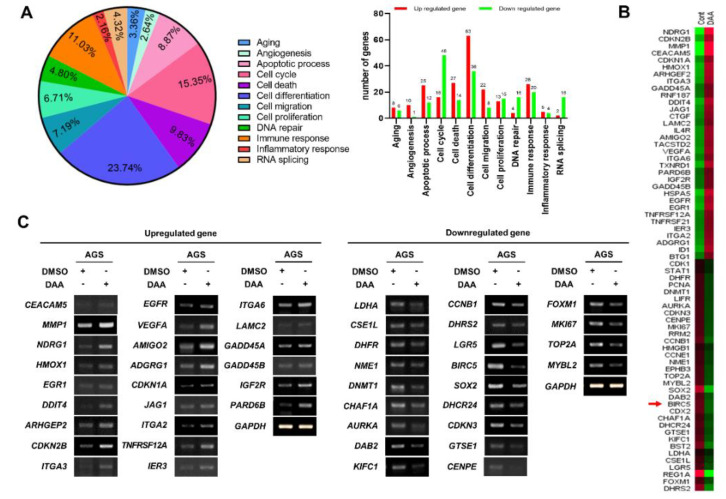
DAA treatment in AGS cells regulated the expression of various genes. (**A**) RNA sequencing was performed on samples prepped from AGS cells that underwent DAA treatment for 48 h. Then, the results of analyzing gene annotation were calculated (upregulated genes: fold change > 1.5, downregulated genes: fold change < 1.5, DAA/DMSO). (**B**) The heatmap shows 33 upregulated genes and 33 downregulated genes showing significant differences in expression. (**C**) Results of RT-PCR on genes (24 of the 33 upregulated genes, 22 of the 33 downregulated genes) analyzed via a heatmap.

In previous studies, we demonstrated that DAA treatment inhibited the proliferation of gastric cancer cells. Then, we wanted to determine which genes were involved in the inhibitory effect on tumor growth. RNA-Seq was performed to identify the genes regulated by DAA treatment. Gene ontology (GO) analysis was performed on the basis of RNA-Seq results. Cell differentiation accounted for the highest percentage (23.74%), followed by cell cycle (15.35%), cell death (9.83%), and apoptotic process (8.87%) related to cell proliferation. The number of upregulated and downregulated genes in each gene category is shown in the right panel (Figure 2A). Among the genes belonging to cell proliferation, apoptosis, and cell cycle from the group showing significant difference in gene expression, 33 genes that are upregulated and 33 genes that are downregulated were selected for heatmap analysis (Figure 2B). The results of GO analysis of 66 genes that were upregulated or downregulated are summarized in Table 1 and Table 2. The upregulated and downregulated genes analyzed in the heat map were analyzed using RT-PCR. As a result, the expression of 24 genes were increased among the 33 upregulated genes. In addition, 22 of the 33 downregulated genes were reduced in expression (Figure 2C). Interestingly, RT-PCR results showed a significant decrease in *BIRC5*, an anti-apoptotic gene. These results suggest that DAA regulates the expression of genes related to cell proliferation.

### 2.3. DAA Treatment Induced Downregulation of mRNA and Protein Survivin Expression

**Figure 3:**
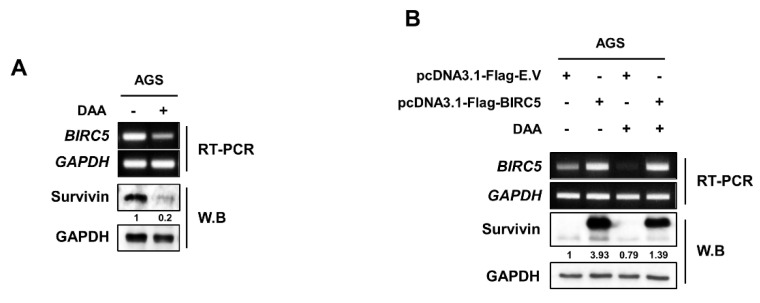
DAA specifically reduced the expression level of survivin. (**A**) Detection of mRNA and protein expression of survivin in AGS cells with 85 µM DAA treatment for 48 h using by RT-PCR and Western blotting. (**B**) The overexpression of survivin and the inhibitory effect of DAA were determined. Survivin was overexpressed in AGS cells using a plasmid system; survivin-overexpressing AGS cells were treated with 85 µM of DAA for 48 h.

We demonstrated that DAA effectively inhibited survivin expression through RNA-Seq results. Therefore, we confirmed DAA could be a potential inhibitor of survivin in cancer therapy. First, AGS cells were treated with DAA, and survivin expression was confirmed by RT-PCR and Western blotting (Figure 3A). The expression level of survivin was significantly decreased by DAA treatment in both mRNA and protein. Even when the survivin gene was overexpressed, the inhibitory effect of DAA was confirmed. DAA was treated to AGS cells overexpressing the survivin gene. Then, the expression was determined by RT-PCR and Western blotting (Figure 3B). The level of *BIRC5* overexpression did not decrease; however, the protein (survivin) expression level also decreased in the overexpressed group. These results suggest that DAA reduces the basal level expression of survivin as well as the expression of overexpressed proteins.

### 2.4. DAA Exhibited a Better Inhibitory Effect on Survivin Than YM-155

**Figure 4:**
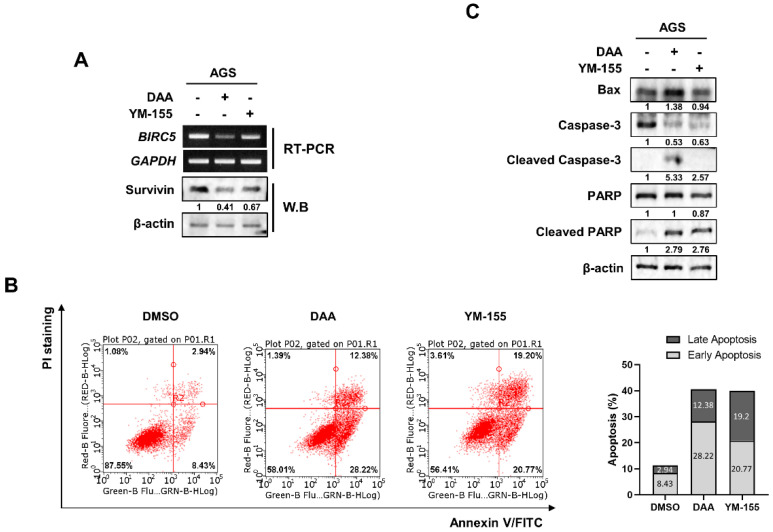
DAA exhibited a better inhibitory effect than survivin inhibitor YM-155. (**A**) AGS cells were treated with 85 µM and 5 nM of DAA and YM-155 for 48 h, respectively, and the expression of survivin was determined by RT-PCR and Western blotting. (**B**) AGS cells were treated with 85 µM and 5 nM of DAA and YM-155 for 48 h, respectively. After annexin V/PI staining, flow cytometry was performed. Bar graph shows the rates of early-stage and late-stage apoptosis. (**C**) AGS cells were treated with DAA and YM-155 for 48 h. Apoptosis-related protein expression was confirmed through Western blotting.

We compared the inhibitory effects of DAA and YM-155 on survivin expression. YM-155 is known to be a survivin inhibitor. AGS cells were treated with DAA and YM-155, and survivin expression was determined by RT-PCR and Western blotting. The data showed that DAA exhibited a better inhibitory effect on survivin than YM-155 (Figure 4A). We then performed flow cytometry to investigate the variation in apoptosis according to the decrease in survivin levels. Our results showed that both DAA and YM-155 had similar apoptotic effects. However, DAA had a high rate of early-stage apoptosis, and YM-155 had a higher rate of late-stage apoptosis (Figure 4B). Additionally, we performed Western blotting to confirm the differences in the expression of proteins related to apoptosis. (Figure 4C). DAA treatment increased the expression of the pro-apoptotic protein BAX. In addition, the levels of cleaved caspase-3 and cleaved poly(ADP-ribose) polymerase (PARP) also increased when treated with DAA compared to YM-155 treatment. These results suggest that DAA has a better inhibitory effect than the survivin inhibitor YM-155.s.

## 3. Discussion

Several anticancer drugs have been used to treat gastric cancer [19]. However, most of these drugs are cytotoxic drugs. Cytotoxic anticancer drugs induce inflammatory reactions. The expression of cytokine proteins caused by these inflammatory reactions is an obstacle to cancer therapy [20]. Therefore, it is important to induce apoptosis in cancer cells. Apoptosis is a process of programmed cell death. Our previous study demonstrated that DAA inhibits gastric cancer cell proliferation by inducing apoptosis [8]. However, we did not know how DAA inhibits the proliferation of gastric cancer cells through apoptosis. Therefore, we performed RNA-Seq to determine which genes were regulated to inhibit cell proliferation. Considerable differences in the expression of genes related to cell death and cell cycle were observed. Additionally, we performed RT-PCR to verify the RNA-Seq results. The RNA-Seq and RT-PCR results were mostly consistent. On the basis of these results, we focused on the inhibition of survivin, a known apoptosis inhibitor.

Survivin belongs to the family of inhibitors of apoptosis (IAP). IAPs are a group of proteins that have endogenous inhibitory roles in apoptosis (programmed cell death) [21]. Our previous study demonstrated that DAA induces apoptosis. However, it is not known whether DAA regulates genes associated with apoptosis. We determined that DAA significantly reduced the mRNA expression and protein expression of survivin.

Survivin is overexpressed in most cancers and has a poor prognosis [22]. We overexpressed survivin in AGS cells to verify that DAA is a specific inhibitor of survivin. DAA treatment of survivin-overexpressing AGS cells showed a significant decrease in protein expression. After survivin overexpression, DAA treatment showed no significant difference in expression at the mRNA level, but significantly decreased the level of protein expression.

YM-155 is a specific survivin inhibitor. However, the clinical trial was discontinued because of its high toxicity and low antitumor effects [18]. In this study, we compared the ability of YM-155 and DAA to reduce survivin expression and functions. At first, we confirmed the effect of survivin expression and function using the same concentration of specific survivin inhibitor and DAA in gastric cancer cells. DAA had a better inhibitory effect on survivin than YM-155 at both the mRNA and protein levels. We also compared the expression levels of proteins that regulate apoptosis. Apoptosis is a signaling process in which caspase proteins are sequentially cleaved and activated. The pro-apoptotic protein BAX was increased by apoptosis. Caspase-3 is cleaved by apoptotic signals. The cleaved caspase-3 cleaves PARP, which is involved in DNA repair [23]. Cleaved PARP does not affect DNA repair. As a result, apoptosis is induced because DNA repair is impossible [24]. Differences between DAA and YM-155 apoptosis-regulating proteins have been demonstrated. BAX levels increased upon treatment with DAA, and the cleaved form of PARP also increased. The active form of caspase-3 was detected only in the DAA-treated group. We determined the difference in the protein expression of caspase signaling and quantified apoptosis. Morphological modification of cell apoptosis is exposed to the outside due to increased phosphatidylserine (PS) in the outer cell membrane [25]. DAA had a higher rate of inducing the early apoptotic stage than YM-155, but YM-155 had a higher rate of inducing the late apoptotic stage. These results showed that there was no significant difference in the total apoptosis rate, but there was a difference in the stage at which apoptosis was induced.

Additionally, we expected that there would be genes specifically regulated by DAA. RNA-Seq results revealed genes that were significantly regulated by DAA treatment. As a result, we attempted to verify that DAA is a specific inhibitor of survivin, a known apoptosis inhibitor. DAA significantly reduced survivin gene and protein expression; in addition, it showed a higher reduction effect than YM-155, a survivin inhibitor. Although DAA had a better inhibitory effect on survivin than YM-155, and it significantly regulated the expression of apoptosis-related proteins, the results of apoptosis quantification were mostly similar.

In conclusion, our study suggests that treatment of DAA in gastric cancer cells regulates proliferation and apoptosis. However, the mechanism by which DAA regulates survivin is unknown, suggesting that further study is needed. Nevertheless, DAA has a better inhibitory effect than YM-155, which is known as a survivin inhibitor, suggesting that it can be developed as a survivin-specific drug in the future.

## 4. Materials and Methods

### 4.1. Chemicals and Reagents

Dehydroabietic acid (DAA) was purchased from Biopurify Phytochemicals Ltd. (Chengdu, China). Rabbit polyclonal antibodies against survivin and GAPDH (A531) were purchased from Bioworld Technology, Inc. (Bloomington, MN, USA). Poly(ADP-ribose) polymerase (PARP) was obtained from GeneTex (Irvine, CA, USA). BAX was purchased from Proteintech, (Rosement, IL, USA). Mouse monoclonal antibodies against Caspase-3 and β-actin were purchased from Santa Cruz Biotechnology (Dallas, TX, USA).

### 4.2. Cell Culture

Human gastric cancer (AGS, MKN-28, SNU-216, SNU-601, SNU-668, and YCC-2) cell lines were obtained from the Korea Cell Line Bank (KCLB, Seoul, Korea). The cell lines were cultured in RPMI-1640 medium supplemented with 5% fetal bovine serum (Corning, NY, USA) and 1% penicillin/streptomycin (Gibco, Waltham, MA, USA) at 37 °C in a 5% CO_2_ atmosphere.

### 4.3. Cell Proliferation Assay

Six human gastric cancer cells were seeded into 96-well plates (1 × 10^4^ cells per well) and incubated for 24 h. Then, the cells were treated with DAA for 24 or 48 h, followed by addition of WST-8 solution (Daeil, Seoul, Korea) and incubated for 30 min. After 30 min of incubation, the plate was shaken gently, and absorbance was measured at 450 nm wavelength using a microplate reader (TECAN, Mannedorf, Switzerland).

### 4.4. Library Preparation and RNA Sequencing

AGS cells were treated with DAA for 48 h, and total RNA was purified using RNAiso Plus reagent (Takara, Shiga, Japan) according to the manufacturer’s instructions. RNA-Seq was commercially commissioned by Ebiogen (EBIOGEN, Seoul, Korea). Total RNA quality was measured using an Agilent’s 2100 bioanalyzer. Library construction was performed using the Quant-Seq library prep kit (Lexogen, Vienna, Austria). Next, we evaluated high-accuracy sequencing with high-throughput single-end 75 bp sequencing using NextSeq 500 (Illumina, San Diego, CA, USA). After sequencing, the differences in gene expression were analyzed using Exdega (EBIOGEN, Seoul, South Korea). For the identification of DEGs, data were normalized and quantified using the LPEseq package [26]. Gene classification was based on searches performed by DAVID (david.abcc.ncifcrf.gov (accessed on 16 December 2020)) and Medline databases (ncbi.nlm.nih.gov (accessed on 12 January 2021)).

### 4.5. Construct of Survivin Overexpression Plasmids

Human survivin plasmids were transfected using Lipofectamine 2000 reagent (Invitrogen, Carlsbad, CA, USA) following the manufacturer’s protocol. Human survivin was cloned using pcDNA3.1-FLAG_BIRC5. The survivin open reading frame (ORF) site was cloned into the pGEM-T Easy vector plasmid within the BamHI and XhoI restriction enzyme sites, thereby creating an expression plasmid construct. Genomic DNA was PCR-amplified using the primers. Genomic DNA was isolated from the AGS cells.

### 4.6. Annexin V-FITC/PI Apoptosis Assay

AGS cells were cultured in 100 mm dishes, followed by overnight incubation with DAA and YM-155 treatment. The cells were collected by trypsinization, washed with DPBS, suspended in 100 µL of binding buffer, and stained with 2 µL of annexin V-FITC (BD Pharmingen, San Jose, CA, USA) and 2 µL (2 mg/mL) of PI (Sigma Aldrich, St. Louis, MO, USA) for 15 min at 25 °C in the dark. Analysis was performed using Guava easyCyte (Luminex, Austin, TX, USA) with 10,000 events each time. Data were analyzed using guavaSoft 3.3 (BD Biosciences, San Jose, CA, USA).

### 4.7. RNA Isolation, cDNA Synthesis, and Polymerase Chain Reaction (PCR)

Total RNA was isolated from AGS cells using RNAiso Plus reagent (Takara, Shiga, Japan) according to the manufacturer’s instructions. cDNA was synthesized from the extracted RNA using a cDNA synthesis kit (TOYOBO, Osaka, Japan) according to the standard protocol. Reverse transcription PCR was performed as described previously [8]. Primers were designed with the following sequences (5′ to 3′): survivin (*BIRC5*) (forward) TGACGACCCCATAGAGGAACA, (reverse) TCAATCCATGGCAGCCAGC, and GAPDH (forward) GGCTGCTTTTAACTCTGGTA, (reverse) ACTTGATTTTGGAGGGATCT.

### 4.8. Western Blotting

Cell lysates were prepared in radioimmunoprecipitation assay buffer (RIPA) buffer (1% NP-40, 0.1% sodium dodecyl sulfate (SDS), 0.5% deoxycholate, 150 mM NaCl, 50 mM Tris (pH 7.5)) containing a protease inhibitor cocktail. Total protein (40 µg) was resolved on SDS-polyacrylamide gels and transferred to polyvinylidene difluoride membranes. The membranes were blocked in 5% skim milk and incubated overnight at 4 °C with primary antibodies diluted 1:1000 in 5% bovine serum albumin (BSA; Bovogen, Keilor East, Australia). Anti-mouse or anti-rabbit HRP-conjugated secondary antibodies were diluted 1:5000 in PBST, and the membranes were incubated in these secondary antibodies for 2 h at 25 °C. Anti-rabbit and anti-mouse polyclonal immunoglobulins were purchased from Bethyl Laboratories (Montgomery, TX, USA). Membranes probed with primary and secondary antibodies were detected using ECL solution (Bio-Rad, Hercules, CA, USA) and Supernova-Q1800 ChemiDoc (Lugen™, Bucheon, Korea) according to the manufacturer’s instructions. Quantitative analysis of Western blot data from more than three experiments was performed with ImageJ and was expressed as mean ± SD

### 4.9. Statistical Analysis

Data are presented as mean ± SEM of five experiments. Fisher’s exact test was used for the statistical evaluation of the results. A *t*-test was used for the statistical analysis of the protein quantification results. All *p*-values were two-sided and were precise. All statistical analyses were performed using GraphPad Prism version 8 (GraphPad Software, San Diego, CA, USA).

## Figures and Tables

**Table 1:** Genes that are upregulated by DAA treatment in AGS cells.

Gene Symbol	Accession Number	Gene Name	Fold Change	*q*-Value
**Cell cycle**				
*CDKN2B*	NM_004936	cyclin-dependent kinase inhibitor 2B	2.484	0.0190
*CDKN1A*	NM_001220777	cyclin-dependent kinase inhibitor 1A	2.095	0.0977
*GADD45A*	NM_001924	growth arrest and DNA damage inducible alpha	1.701	0.3246
*PARD6B*	NM_032521	par-6 family cell polarity regulator beta	1.621	0.4235
**Cell proliferation**				
*HMOX1*	NM_002133	heme oxygenase 1	2.661	0.0094
*ARHGEF2*	NM_004723	Rho/Rac guanine nucleotide exchange factor 2	2.493	0.0184
*EGFR*	NM_005228	epidermal growth factor receptor	2.278	0.0426
*VEGFA*	NM_001171625	vascular endothelial growth factor A	2.175	0.0969
*JAG1*	NM_000214	jagged 1	2.094	0.0871
*ITGA2*	NM_002203	integrin subunit alpha 2	1.966	0.1823
*BTG1*	NM_001731	B-cell translocation gene 1, anti-proliferative	1.781	0.2930
**Apoptosis**				
*HSPA5*	NM_005347	heat shock protein family A (HSP70) member 5	2.806	0.0138
*AMIGO2*	NM_181847	adhesion molecule with Ig-like domain 2	2.116	0.1138
*IER3*	NM_003897_4	immediate early response 3	1.838	0.2725
*IGF2R*	NM_000876	insulin-like growth factor 2 receptor	1.665	0.4105
**DNA replication**				
CTGF	NM_001901	connective tissue growth factor	1.936	0.1439

**Table 2:** Genes that are downregulated by DAA treatment in AGS cells.

Gene Symbol	Accession Number	Gene Name	Fold Change	*q*-Value
Cell cycle				
CDK1	NM_001170406	cyclin-dependent kinase 1	0.657	0.3084
CCNE1	NM_001238	cyclin E1	0.559	0.1132
AURKA	NM_198434	aurora kinase A	0.537	0.0878
CDKN3	NM_001130851	cyclin-dependent kinase inhibitor 3	0.527	0.0754
GTSE1	NM_016426	G2 and S-phase expressed protein 1	0.524	0.0695
CENPE	NM_001813	centromere protein E	0.524	0.0696
MKI67	NM_001145966	marker of proliferation Ki-67	0.512	0.0813
KIFC1	NM_002263	kinesin family member C1	0.486	0.0359
CCNB1	NM_031966	cyclin B1	0.451	0.0168
Cell proliferation				
STAT1	NM_007315	signal transducer and activator of transcription 1	0.657	0.3363
NME1	NM_198175	NME/NM23 nucleoside diphosphate kinase 1	0.559	0.1130
LIFR	NM_001127671	leukemia inhibitory factor receptor alpha	0.547	0.0905
FOXM1	NM_001243088	forkhead box M1	0.521	0.0652
DHCR24	NM_014762	24-dehydrocholesterol reductase	0.496	0.0756
CDX2	NM_001265	caudal type homeobox 2	0.444	0.0173
DHRS2	NM_005794	dehydrogenase/reductase (SDR family) member 2	0.356	0.0023
SOX2	NM_003106	SRY-box 2	0.264	0.0001
REG1A	NM_002909	regenerating family member 1 alpha	0.144	0.0001
Apoptosis				
HMGB1	NM_002128	high mobility group box 1	0.621	0.2869
BIRC5	NM_001168	baculoviral IAP repeat containing 5	0.449	0.0162
DNA replication				
PCNA	NM_182649	proliferating cell nuclear antigen	0.55	0.0937
CHAF1A	NM_005483	chromatin assembly factor 1 subunit A	0.538	0.0852
TOP2A	NM_001067	topoisomerase (DNA) II alpha	0.511	0.0885
RRM2	NM_001165931	ribonucleotide reductase regulatory subunit M2	0.507	0.0482

## Data Availability

The data presented in this study are available within the article.
